# NAFLD mark: an accurate model based on microRNA-34 for diagnosis of non-alcoholic fatty liver disease patients

**DOI:** 10.1186/s43141-021-00257-5

**Published:** 2021-10-18

**Authors:** Amal A. Mohamed, Ahmed El-Demery, Eman Al-Hussain, Shroouk Mousa, Ahmed Abdel Halim, Sahar M. Mostafa, Reda S. Abdelghany, Seham M. Mahmoud, Mohammad A. Elkady, Khaled Raafat, Alshymaa A. Hassnine, Mohamed M. Omran

**Affiliations:** 1 Biochemistry Department, National Hepatology and Tropical Medicine Research Institute, Cairo, Egypt; 2 Biochemistry Department, Faculty of Medicine, October 6 University, 6th of October City, Egypt; 3 Clinical and Chemical Pathology, Faculty of Medicine, Cairo university, Giza, Egypt; 4 Internal Medicine Department, Faculty of Medicine, Cairo University, Cairo, Egypt; 5 Tropical Department, National Hepatology and Tropical Medicine Research Institute, Cairo, Egypt; 6 Tropical Medicine Department, Ahmed Maher Teaching Hospital, Cairo, Egypt; 7 Tropical Department, El-Sahel Teaching Hospital, Cairo, Egypt; 8 Theodor Bilharz Research Institute Gastroenterology and Hepatology department, Cairo, Egypt; 9 Gastroenterology and Hepatology Department, Faculty of Medicine, Ain Shams University, Cairo, Egypt; 10 Department of Gastroenterology and Tropical Medicine, Faculty of Medicine, Minia University, Minia, Egypt; 11 Chemistry Department, Faculty of Science, Helwan University, Ain Helwan, Cairo, 11795 Egypt

**Keywords:** Non-alcoholic fatty liver disease, MicroRNA-34, Alanine aminotransferase, Body mass index, Cholesterol, C-reactive protein

## Abstract

**Background:**

It remains essential for non-alcoholic fatty liver (NAFLD) patients, to develop a sensitive and specific diagnostic model. Data regarding the use of micro (mi)RNA-34 for NAFLD diagnosis are few. Routine clinical assessment, laboratory tests were done for Egyptian individuals (*n* = 314) were included (100 healthy individuals and 214 NAFLD patients). Quantification of miRNA-34 was done using real-time PCR. Extremely significant variables were entered into stepwise logistic regression. The diagnostic power of variables was estimated by the area under the ROC (AUC).

**Results:**

MiRNA-34 levels were higher in NAFLD patients than healthy individuals with a significant difference (*P*< 0.0001). The multivariate analysis was used to evaluate the NAFLD-associated variables (CRP, cholesterol, body mass index (BMI), ALT had *p*< 0.0001 while mRNA-34 had (*p*=0.0004). The AUCs (CI) of candidate NAFLD markers were in the order of miRNA-34 0.72 (0.66–0.77) < ALT 0.73 (0.67–0.79) < BMI 0.81 (0.76–0.86) < cholesterol < 0.85 (0.79–0.90) < CRP 0.88 (0.84–0.92). We developed a novel index for discriminating patients with NAFLD named NAFLD Mark. AUC was jumped to 0.98 (0.93–0.99) when five markers were combined. The AUC of NAFLD mark for NAFLD detection was higher than the AUCs of seven common NAFLD indexes (0.44–0.86).

**Conclusions:**

The NAFLD mark is a non-invasive and highly sensitive and specific model for NAFLD diagnosis.

## Impact statement

Clinical, biochemical, and imaging tools were used for the early diagnosis of NAFLD. A liver biopsy had several disadvantages such as invasive and expensive so there is a need for sensitive and specific tests. The current work aimed to develop a novel model for NAFLD diagnosis using miRNA-34 and routine laboratory parameters

## Background

NAFLD is a silent killer disease that is characterized by high hepatic fat aggregation especially aggregation of triglyceride [1]. NAFLD incidence was 25% in the overall population and about 90% among obese patients [2]. The incidence of NAFLD in Egypt is about 16% [3]. NAFLD may lead to chronic liver diseases (hepatic cirrhosis and hepatocellular carcinoma [4]. NAFLD diagnosis is the first step for the evaluation of NAFLD severity [5]. Liver biopsy is the basic tool for NAFLD evaluation, but in routine clinical diagnosis, it has several disadvantages (invasive, risk, and expensive). There is a demand for sensitive, and specific, and non-invasive variables. Clinical and laboratory investigations are used in NAFLD diagnosis [6]. There are several models were developed to evaluate hepatic NAFLD [7]. The fatty liver index (FLI) has an AUC of 0.84 [7]. The hepatic steatosis index (HSI), liver fat score, SteatoTest, and PLALA had AUC of 0.81, 0.86, 80, and 0.86, respectively [8–11]. The fibrosis-4 (FIB-4) includes age, liver enzymes (AST, ALT), and platelet count [12]. BAAT is based on BMI, age, ALT, and triglycerides [10, 13].

Application of miRNAs (miRNA-29a, miR-34a, and miRNA-122) for NAFLD diagnosis is of particular interest, among which miR-34a, is the most associated with NAFLD development. MiR-34a has a significant role in increasing lipid synthesis and inhibiting mitochondrial fatty acid oxidation in hepatocytes and leading to altered lipid metabolism in NAFLD. miR-34a inhibits gene which regulates catabolism [14, 15]. Therefore, this work aimed to develop a novel model for NAFLD diagnosis using miRNA-34 and clinical and routine laboratory markers. Furthermore, we aim to validate the diagnostic accuracies of seven non-invasive models in comparison with the NAFLD mark for NAFLD diagnosis

## Patients and methods

### Patients

This case-control study was included healthy individuals (*n*=100) and NAFLD patients (*n*=214). All individuals were diagnosed by abdominal ultrasound and FLI score according to Bedogni et al. [16]. Written consent was obtained from all subjects. Exclusion criteria: patients with liver or kidney chronic diseases, and alcohol intake. Full history and clinical examination (BMI and waist circumference) were taken from all patients.

### Laboratory investigation

The fasting blood sample (10 ml) was withdrawn from all individuals and divided into three parts: the first part treated with sodium citrate for INR–prothrombin determination, the second part treated with EDTA for complete blood count, and the third part without blood coagulant for evaluation biochemical parameters. Routine biochemical tests were done by automated chemistry analyzer OLYMPUS AU 400 (Olympus America, Pennsylvania, USA). C-reactive protein (CRP) was done using nephelometry kit, CA, USA; alfa fetoprotein (AFP) was measured using CanAg AFP EIA 600-10 and 25 OH D using (EIA-5396; DRG International Inc., Springfield., New Jersey, USA) according to the manufacturer’s instructions.

### Determination of the miR-34 level

Serum samples were stored at −80°C until assayed and thawed immediately before the miRNA-34 determination. Firstly, the total RNA was extracted and purified using miRNeasy Mini Kit (Qiagen, Hilden, Germany) according to the manufacturer’s procedures. cDNA was synthesized by reverse transcription reaction using TaqMan MicroRNA (Applied Biosystems, Foster City, USA) and the thermal cycler (Quanta Biotech). The miR-34 level was amplified from cDNA using TaqMan Universal Master Mix and TaqMan assay (Catalog no: 4427975). The RNU49 was used as a housekeeper gene (Cat no: PN4427975; ID: 001005). All samples were analyzed using the 5 plex Rotor-Gene PCR Analyzer (Qiagen, Germany). The 2^ΔΔCt^ method was conducted for the analysis of gene expression levels using TaqMan microRNA Control Assays RNU49 for normalization purposes. Threshold cycle (CT) was calculated Δ CT value, using the formula [ΔΔ CT = Δ CT_tumor_−Δ CT_normal_]; the fold change was calculated as following [FC= 2^- ΔΔ CT^] and then log FC was calculated [17].

### Statistical analysis

Statistical analysis was achieved using the SPSS statistical and graph pad programs. To analyze the normal distribution of parameters in both groups, the Kolmogorov–Smirnov test was applied. Parametric data were presented as the mean and standard deviation (SD) while non-parametric data were presented as median or interquartile range. Student *t* test, Mann-Whitney, and chi-square test were used for data analysis. Univariate analysis was done using Student *t* test, Mann-Whitney, and chi-square test in addition to multivariate analysis were carried out to screen the independent risk factors of NAFLD. The diagnostic value of each variable was assessed by plotting the receiver operating characteristic curve (ROC curve) and determine the area under the curve (AUC). We determined the variable best cut-off value for NAFLD diagnosis (maximum value for the sum of sensitivity and specificity). The diagnostic indexes were expressed as a percentage. Variables with a *p* value <0.05 at multivariate analyses and high area under ROC were entered into stepwise logistic regression. Common NAFLD indexes (PLALA score [11], FIB-4 [18], BARD [19], NAFLD fibrosis score [12], BAAT [10, 13], AAR [20], and APRI were calculated as original papers [21]).

## Results

### Patient characteristics

**Fig. 1:**
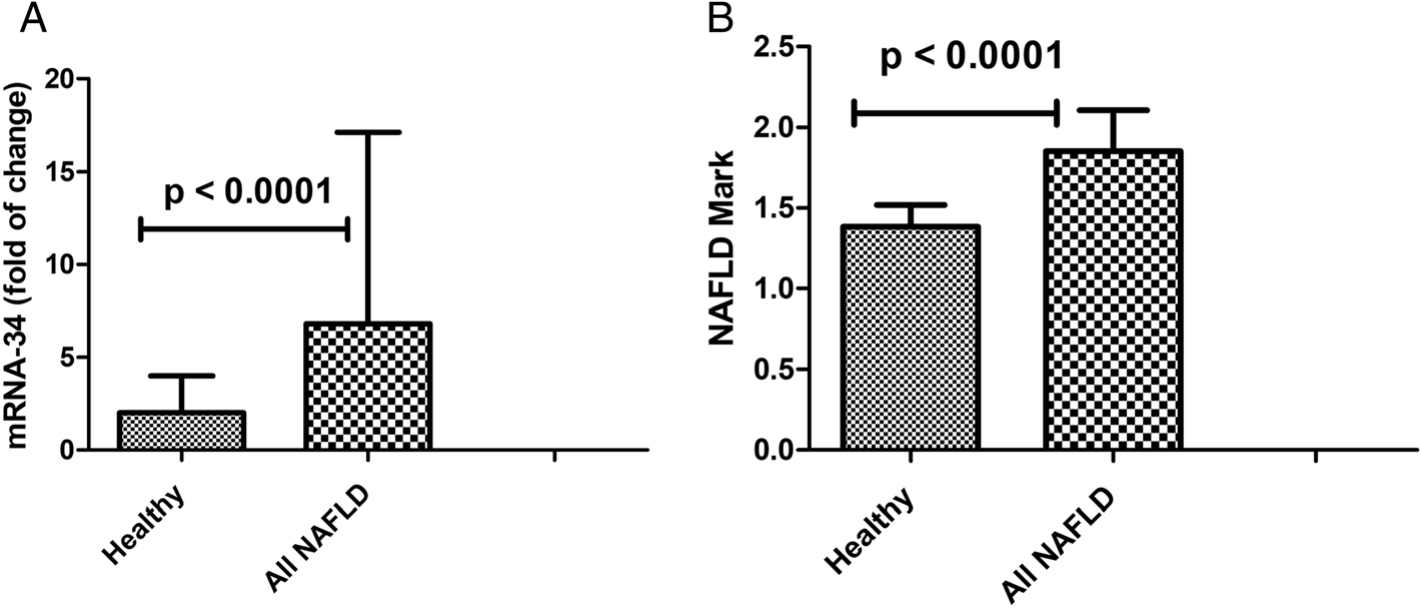
Level of miRNA-34 and NAFLD mark. **A** Level of miRNA-34 in healthy and NAFLD groups. **B** Level of NAFLD mark in healthy and NAFLD groups. Error bar is SD

The clinical and laboratory data of the two studied groups were presented in Table 1. MiRNA-34 levels were significantly increased (*p*< 0.0001) in NAFLD patients than in healthy individuals (Fig. 1A). Using multivariate analysis showed that the increase in miRNA-34 levels was significant (*p*=0.004). CRP, cholesterol, ALT, and BMI variables were significant (*p*< 0.0001) associated with the presence of NAFLD.

**Table 1:** Demographic and laboratory features of the studied groups

Parameter	Univariate analysis			Multivariate analysis	
	Healthy (*n*=100)	All NAFLD (*n*= 214)	*p* value	Odds ratio (95% CI)	*p* value
Gender					
Male (no, %)	52 (52%)	106 (49.5%)	0.38	-	-
Female (no, %)	48 (48%)	108 (50.5%)			
Age (years)	54.1±15.8	52.8 ± 10.5	0.39	-	-
BMI (kg/m^2^)	23.7±4.3	30.2±5.9	< 0.0001	2.8 (1.7–3.5)	**0.001**
Hemoglobin (g/L)	113±18	113±18	0.97	-	-
Glucose (mg/dl)	100±16	110±13	< 0.0001	1.7 (1.2–1.9)	0.007
Alanine aminotransferase (U/L)	30.3 ±5.1	35.2± 7.2	< 0.0001	2.6 (1.6–2.9)	**< 0.0001**
Aspartate aminotransferase (U/L)	33.1±7.4	37.3±8.0	< 0.0001	1.2 (0.98–1.1)	0.097
Gamma-glutamyl transferase (U/L)	34.0±9.1	43.0±16.8	< 0.0001	0.93 (0.9–1.0)	0.43
Albumin (g/L)	40.2 ±2.1	38.3±2.1	0.09	-	-
Total bilirubin (mg/dl)	0.77±0.2	0.86±0.3	< 0.0001	0.2 (0.0–0.76)	0.55
C-reactive protein (mg/L)	2.1 (2–3)	6.5 (4–12)	< 0.0001	4.5 (2.6–7.0)	**< 0.0001**
Alfa fetoprotein (U/L)	6.0 (5–7)	7.0 (5–9)	< 0.0001	1.6 (1.1–1.7)	0.01
International normalized ratio-PT	0.98±0.1	1.1±0.2	< 0.0001	0.92 (0.1–1.1)	0.44
Cholesterol (mg/dl)	142.8±22.0	181.5±27.0	< 0.0001	3.1 (2.5–4.9)	**< 0.0001**
Triglycerides (mg/dl)	141.6±17.7	168.1±34.5	< 0.0001	1.6 (1.2–1.8)	0.01
High-density lipoprotein (mg/dl)	42.5±0.8.3	38.6±9.3	< 0.0001	0.93 (0.84–1)	0.18
Low-density lipoprotein (mg/dl)	104.4±10.8	115.0±23.2	< 0.0001	1.5 (1.2–1.9)	0.03
Vitamin D (pmol/L)	88 (77–116)	52 (34–78)	< 0.0001	0.98 (0.95–1.1)	0.151
MiRNA-34 (fold of change)	2.0 (1.4–4.0)	6.8 (2.0–32)	< 0.0001	2.1 (1.5–2.3)	**0.0004**

### Diagnostic performance of miRNA-34and candidate’s markers

The diagnostic power of single (CRP, cholesterol, BMI, ALT, and miRNA-34) to diagnose NAFLD was evaluated using area under ROC and presented in Table 2. Variables (CRP, cholesterol, BMI, ALT, and mRNA-34) with a *p* value <0.05 at multivariate analyses and high AUCs were entered into the stepwise logistic regression. CRP is the most efficient index among clinical and laboratory variables. So, CRP was the best variable to combine with other variables to discriminate against NAFLD patients. Thus, each variable provides independently different information and therefore was expected to increase the diagnostic performances if five variables were combined for the detection of NAFLD. The best linear combinations of blood markers were selected by stepwise multi-discriminant analysis for the development of a novel model (NAFLD mark) based on five markers (CRP, cholesterol, BMI ALT, and miRNA-34). NAFLD mark = [(CRP (mg/L) × 0.025) + cholesterol (mg/dl × 0.005 + BMI (kg/m^2^) × 0.013 + (ALT (U/L) × 0.008 + miRNA (FC) × 0.002) + 0.044]. The levels of the model in healthy and NAFLD patients were presented in Fig. 1B. The mean [SD] of NAFLD mark studied groups was 1.38 [0.13]; 1.85 [0.25] for healthy individuals, NAFLD with highly significant differences (*p* < 0.0001). The AUC and diagnostic indexes for the NAFLD mark were presented in Table 2. The NAFLD mark can be used equally effectively in gender without significant difference (*p* > 0.05).

**Table 2:** Diagnostic power of single and combined candidate markers (CRP, cholesterol, BMI, ALT, and miRNA34) to diagnose NAFLD

Variables	AUC (95% CI)	*p* value	Cut-off	Sensitivity	Specificity	PPV	NPV
miRNA-34	0.72 (0.66–0.77)	< 0.0001	3.5	70	64	81	49
ALT	0.73 (0.67–0.79)	< 0.0001	32	77	66	83	57
BMI	0.81 (0.76–0.86)	< 0.0001	25	75	67	83	56
Cholesterol	0.85 (0.79–0.90)	< 0.0001	160	83	83	92	67
CRP	0.88 (0.84–0.92)	< 0.0001	3	85	85	92	72
CRP+ cholesterol	0.93 (0.89–0.96)	< 0.0001	1.6	90	85	93	79
CRP+ cholesterol+ BMI	0.94 (0.91–0.97)	< 0.0001	1.4	91	87	95	78
CRP+ cholesterol+ BMI+ALT	0.95 (0.93–0.97)	< 0.0001	1.4	92	88	95	81
CRP+ cholesterol+ BMI+ALT+ miRNA-34	0.98 (0.93–0.99)	< 0.0001	1.5	93	90	95	86

### Evaluation of NAFLD mark vs non-invasive models for NAFLD

**Fig. 2:**
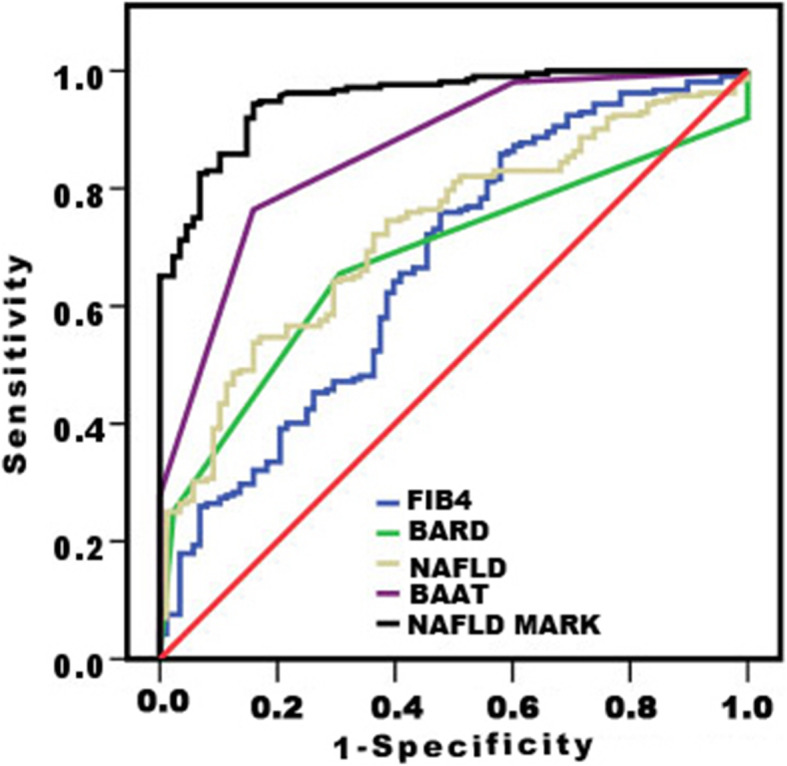
Evaluation of diagnostic performance of NAFLD mark vs non-invasive models for NAFLD using area under ROC curve

The AUC data and the diagnostic performances of seven NAFLD indexes (AAR, PALA, APRI, FIB4, BARD, NAFLD, and BAAT) were compared to NAFLD diagnosis (Table 3, Fig. 2). The NAFLD mark was the most efficient index for NAFLD diagnosis.

**Table 3:** Evaluation of NAFLD mark vs non-invasive models for NAFLD

Index	AUC (95% CI)	*p* value	Cut-off	Sensitivity	Specificity	PPV	NPV
AAR	0.44 (0.37–0.51)	0.68	1.0	56	64	77	41
PALA	0.48 (0.41–0.55)	0.51	1.0	9	98	90	33
APRI	0.58(0.51–0.64)	0.19	0.35	59	50	72	36
FIB4	0.66 (0.60–0.73)	0.03	1.2	59	62	76	41
BARD	0.68 (0.62–0.74)	0.001	2.0	66	67	81	49
NAFLD	0.73 (0.66–0.78)	< 0.0001	1.0	51	85	88	45
BAAT	0.86 (0.82–0.91)	< 0.0001	2.0	72	100	100	63
NAFLD mark	0.98 (0.93–0.99)	< 0.0001	1.5	93	90	95	86

## Discussion

NAFLD is an over-accumulation of triglyceride in the liver without alcohol consumption. NAFLD can develop chronic liver diseases and hepatic cancer. Liver biopsy is the principal tool with several complications (expensive, invasive with high sampling error). There is a demand for accurate, and non-invasive models and existing non-invasive NAFLD models are inadequate [22]. Diagnosis of NAFLD using various clinical and laboratory data, scoring systems, and imaging methods (abdominal ultrasonography and computed tomography) has been evaluated [23]. The sensitivity of these variables is low. We evaluated several variables to develop the NAFLD mark for NAFLD diagnosis. The NAFLD mark included CRP, cholesterol, BMI, ALT, and miRNA-34. BMI is one of the most variables for obesity evaluation and correlated with the incidence of NAFLD. Higher BMI was associated with the incidence of NAFLD (4–14 fold increase) [24]. BMI was the third diagnostic power variable in the NAFLD mark. This result agrees with the assumption that obesity is the main accountable for NAFLD [2]. The macrophages in fatty cells secrete proinflammatory cytokines such as CRP, and cytokines which impair insulin signaling, inducing insulin resistance. CRP has also been a biomarker of the NAFLD scoring system and a strong predictor of NAFLD. In NAFLD patients, ALT level is accompanying with liver necrosis and liver damage. ALT levels were within normal in 25% of NAFLD patients [25, 26]. ALT was the best predictor of NAFLD had AUC 0.93 with 94% sensitivity and 72% specificity [27]. MiRNA-34 contributes to liver inflammation through an apoptosis pathway and may be used as a biomarker for diagnosing NAFLD [28]. miRNA are small single-stranded RNA (21 to 23 nucleotides) that are responsible for regulating gene expression [29]. In the present study, there was a significant increase (*p*< 0.0001) in miRNA-34 levels in NAFLD patients than healthy individuals with an AUC of 0.72 for NAFLD diagnosis. The diagnostic power of miR-29a was 0.68 AUC with 61% sensitivity and 82% specificity while miRNA-122 had 0.83 AUC with 75% sensitivity and 82% specificity [30]. Both miRNA-122 and mRNA-34a levels were a significant increase in NAFLD (*p*<0.001) than normal individuals. miRNA-122 had 92% sensitivity and 85% specificity to discriminate NAFLD from healthy individuals [31]. Simple models have been evaluated including AAR [20], APRI [21], BARD score [19], FIB-4 [18], PALA [11], NAFLD fibrosis score [11], BAAT index [10, 13], FLI, HSI, and NAFLD-liver fat score (NAFLD-LFS). These simple methods do not reflect the mechanism of the NAFLD directly. A developed model for NAFLD diagnosis based on smoking, obesity, hypertension, cholesterol, triglycerides, and ALT had an AUC of 0.81 [32]. For NAFLD diagnosis, FIB-4 had an AUC of 0.81 and APRI had an AUC of 0.73 [33], and the fatty liver index had AUC ranged from 0.81 to 86 while lipid accumulation product had AUC ranged for 0.77–0.92 [34]. Five NAFLD models were evaluated using AUCs. AUCs were in the order of NAFLD liver fat score had AUC of 0.80< hepatic steatosis index had of 0.81< fatty liver index had of 0.83 < triglyceride × glucose model had of 0.90 < visceral adiposity index had 0.92 [35]. Limitations of this study are a single-center study and recommended the validation of NAFLD mark in multi-center studies.

## Conclusions

NAFLD mark is a highly sensitive and specific model for NAFLD diagnosis. NAFLD mark showed superior and more accurate diagnostic tool than seven NAFLD indexes for NAFLD diagnosis.

## Data Availability

The authors declare that all generated and analyzed data are included in the article.

## Abbreviations

AAR: ALT/AST ratio
ALT: Alanine aminotransferase
APRI: Aspartate aminotransferase to platelet ratio index
AST: Aspartate aminotransferase
AUC: Area under ROC curve
BAAT: BMI, age, ALT, triglycerides index
BARD: Body mass index
AST: ALT, diabetes index
BMI: Body mass index
CI: Confidence interval
CRP: C-reactive protein
CT: Threshold cycle
EDTA: Ethylene diamine tetra-acetic acid
FC: Fold of change
FIB-4: Fibrosis index
FLI: Fatty liver index
GGT: Gamma-glutamyl transferase
HSI: Hepatic steatosis index
INR: International Normalized Ratio
miRNA: MicroRNA
NAFLD: Alcoholic fatty liver
NASH: Nonalcoholic steatohepatitis
NPV: Negative predictive value
PLALA: Platelet count, albumin
PPV: Positive predictive value
ROC curve: Receiver operating characteristic curve
SD: Standard deviation
